# Temporomandibular Joint Disorder and Airway in Class II Malocclusion: A Review

**DOI:** 10.7759/cureus.30515

**Published:** 2022-10-20

**Authors:** Shruti Rathi, Rizwan Gilani, Ranjit Kamble, Sakshi Bhandwalkar

**Affiliations:** 1 Orthodontics and Dentofacial Orthopedics, Sharad Pawar Dental College, Datta Meghe Institute of Medical Sciences, Wardha, IND; 2 Orthodontics, Sharad Pawar Dental College, Wardha, IND; 3 Public Health Dentistry, Sharad Pawar Dental College, Datta Meghe Institute of Medical Sciences, Wardha, IND

**Keywords:** pain, class ii malocclusion, tmj disorders, airway disorders, malocclusion of teeth

## Abstract

In class II malocclusion, there is an anteroposterior disparity between the upper dentition and the lower dentition, which may or may not be accompanied by a skeletal discrepancy. For orthodontists, this is one of the common malocclusions encountered during clinical practice. This might be due to excess maxillary growth or retarded growth of the mandible or a combination of both. In such types of malocclusion, both the upper and lower airways are affected, the lower one most commonly. Characteristic features seen are a narrow maxillary arch, a proclined upper anterior, and mouth breathing as a developing habit. Also, the position of the condyle in the skeletal type of class II malocclusion plays a vital role in the development of temporomandibular joint disorders. Treating such disparity in a growing individual leads to better results in the long term as well as prevention of malocclusion taking a severe form. Myofunctional appliances are useful for repositioning the mandible as well as the condyle. In adults, extraction of the upper premolars is most commonly done for the correction of class II malocclusion. This provides the patient with a better esthetic appearance. In addition to this, various treatment modalities, such as splint therapy, exercise, and prolotherapy, are beneficial for pain relief and temporomandibular disorder (TMD) correction. This article deals with the characteristics, development, etiology, and comprehensive treatment options of class II malocclusion and its co-relation with the upper and lower airway along with the severity of temporomandibular joint disorders. Repositioning of the condyle in the glenoid fossae is the key to the correction of this disorder.

## Introduction and background

Malocclusions of class II type are of interest to professional orthodontists because they account for a considerable proportion of the individuals they treat [1]. Individuals with normal occlusion and skeletal relationship have synchronized maxillary and mandibular growth, resulting in a well-balanced and esthetically pleasing profile, whereas individuals with class II malocclusions have an anteroposterior discrepancy between the maxillary and mandibular dentitions, which may or may not be accompanied by a skeletal discrepancy. Among all the craniofacial disharmonies, skeletal class II is the most frequently associated condition with the narrowing of the upper airway (UA). There seems to be a consensus that maxillary protrusion increases UA length, and mandibular retrusion is related to its constriction.

Vertical craniofacial disharmonies are also related to UA morphology and respiratory function. Also, the position of the condyle plays an important role since it affects the growth of the mandible. It has been observed that skeletal class I and class II individuals showed a “wide” type of airway morphology, with skeletal class II patients having a higher width-to-depth ratio compared to skeletal class I patients. In other words, the airways in class II patients are smaller anteroposteriorly compared to the airways of class I patients. The pharyngeal airway (PA) comprises three parts: the nasopharynx, oropharynx, and hypopharynx.

The nasopharyngeal airway (NA) is a conical tube made up of muscles and mucosa. The adenoid, a complex network of lymphatic tissues located in the posterior area, is also included. Predisposing factors, such as repeated infection or inflammation, usually result in adenoid hypertrophy and posterior airway constriction in growing children. Because of partially impaired nasal respiration function, children with narrowed NAs tend to use mouth breathing. The temporomandibular joint (TMJ) is a complex anatomical structure in the human body with important clinical implications in dentistry. The mandibular condyle is a component of the temporomandibular joint structure, and its volume and shape influence treatment response stability in orthodontic, orthopedic, orthognathic, and prosthodontic patients.

During the treatment procedure, dentists should consider the position and morphology of the condyle. The vertical facial pattern is known to influence maximum occlusal force and masticatory muscle activity. The hyperdivergent group had the lowest condylar inclination with the mid-sagittal plane, indicating that the condyle is located more anteriorly in this group. The vertical condylar inclination, which represents the anterior and posterior condylar inclinations, increased in the hyperdivergent group, indicating more posterior condyle rotation, and decreased in the hypodivergent group, indicating more anterior condyle rotation. It affects the severity of the disorder and thus, the temporomandibular joint. According to the literature, TMD signs and symptoms are more common in class II malocclusion. Knowledge of regular condyle-fossa variations caused by vertical skeletal patterns in patients with skeletal class II mandibular retrognathism could aid in the diagnosis of TMJ pathologies, understanding of TMDs, and consideration during orthodontic treatment. Because the condylar position shifts from anterior to posterior with age, if intervention begins early, the corrections achieved will be more stable as the growth period is utilized.

## Review

Classification of class II malocclusions: In general, they are classified as either dental, skeletal, and/or functional components or characteristics. The various components responsible for this type of malocclusion are mentioned in the following sections.

Characteristics of class II malocclusions with respect to teeth

Angle suggested a classification system that had its foundation on the mandibular first molars' position to the maxillary first molars [1]. He defined class II malocclusions as having a distal connection of more than one-half the width of the cusp between the mandibular teeth and the maxillary teeth. There is a question about using the first molar relationship as the primary criterion for classifying malocclusions because each class of malocclusion contains numerous variations that have a substantial impact on the treatment method. Despite these evident shortcomings, Angle's classification is still extensively utilized as a way of description and communication among dental professionals due to its simplicity.

He characterized two types of class II malocclusions based on the inclination of the upper central incisors. Class II, division 1 malocclusions have labially inclined maxillary incisors, an increased overjet, and a moderately narrow maxillary arch. The overlap of the vertical incisors can range from a severe overbite to an open bite. The excessive lingual inclination of the maxillary central incisors overlapped on the labial by the maxillary lateral incisors is classified as a class II division 2 type of malocclusion. This is frequently associated with more vertical overlap of incisors and minimal horizontal overlap. In cases with an extreme overbite, the incisal edges of the lower incisors may contact the soft tissues of the palate [2]. In a few class II division 2 types of cases, the palatally inclined maxillary incisors may additionally traumatize the mandibular labial gingival tissues, especially in the absence of an overjet. An inverted maxillary occlusal plane has two separate occlusal levels, one in supra occlusion for the front teeth and one in relative infra occlusion for the posterior portions. With extrusion of the mandibular incisors, an exaggerated Spee curve may be found in the mandibular arch.

Characteristics of class II malocclusions with respect to jaw

Most commonly, class II instances with anteroposterior skeletal discrepancies have a high ANB angle and a Wits appraisal, indicating a discrepancy between the maxilla and mandible. Anteroposterior skeletal disparities may be accompanied by a vertical disparity, such as an unusually long or short anterior face. The amount of abnormality is usually observed by comparing the dental and facial appearance of individuals with a given type of malocclusion to another set of individuals with normal occlusion and morphologic connections. Common cephalometric traits may be observed in individuals with the same type of malocclusion, and these characteristics may differ dramatically from those with normal occlusion or other forms of malocclusions.

Significant characteristics of the cephalogram of class II division 1 malocclusion: Using this classification, numerous studies have been performed to elaborate on the characteristics specific to class II division 1 malocclusion. Fisk described the following six possible morphological variations in their dentofacial complex: (1) the maxilla and teeth are anteriorly situated with respect to the cranium, (2) the position of the maxilla is normal while the upper teeth are anteriorly placed, (3) the mandible is normal in size, but posteriorly positioned, (4) the mandible is underdeveloped, (5) the mandibular teeth are posteriorly placed on a mandible that is in a normal position, and (6) various combinations of the above relationships [3].

Perioral functional characteristics of class II malocclusions

Class II malocclusions might be related to abnormal muscle patterns [4]. The larger overjet in class II division 1 permits the lower lip to sit between the upper and lower incisors, preserving the overjet. Also, abnormal muscle activity of mentalis and abnormal buccinator activity, combined with the compensatory position of the tongue and its function, may cause changes in dental and facial structures such as constricted upper jaw posterior segments, protrusion and spacing of the upper incisors, and mandibular incisors inclined abnormally during swallowing [5]. The orbicularis oris and mentalis muscles are frequently well-developed and active in class II division 2 persons [6]. The lingual inclination of the maxillary incisors may enhance the appearance of the lower "lip curl" associated with the vertical incisor closure.

Role of Airway in Class II Malocclusion

The upper airway is a structure that is in possession of one of the most important critical processes in the human body: breathing. The study of the upper airway has always been of interest in orthodontics, with the main goal of clarifying the correlation between pharynx structures and the growth of craniofacial complex and development. Obstructive processes of morphologic, physiologic, or pathologic character, such as adenotonsillar hypertrophy, chronic and allergic rhinitis, irritants of environmental factors, infections, congenital nasal deformities, traumas of the nose, polyps, and tumors, are risk factors for upper airway obstruction. When this occurs, a functional imbalance causes an oral breathing pattern, which can affect facial morphology and dental arch shapes, resulting in malocclusion.

**Figure 1:**
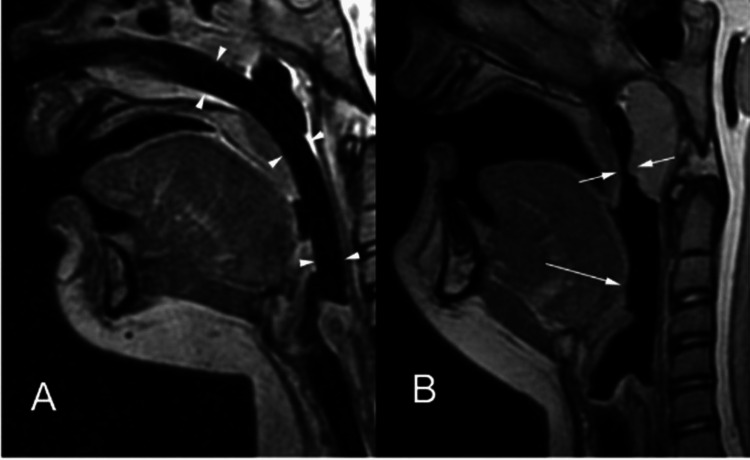
Airway depiction in MRI. MRI accounts for better soft tissue evaluation, especially in the airway. (A) depicts the airway being divided into the nasopharynx, oropharynx, and hypopharynx, whereas (B) depicts a constricted airway.

The literature has reported a relationship between mouth breathing and class II malocclusions, as well as an association between the high-angle pattern of growth and obstruction of the airway concurrently with mouth breathing [7]. If this association exists, class II malocclusions and high-angle growth patterns must be caused by natural anatomical reasons. Among the risk factors for pharyngeal airway obstruction that can be treated include allergies, environmental irritants, and infections. There is also an inherent physical predisposition to narrower airway passage [8], as depicted in Figure 1. As a result, healthy patients with class II malocclusions and vertical development patterns may have narrower airways than normal occlusions and growth patterns or class I malocclusions. In class II malocclusions with no evident pharyngeal pathology, the nasopharynx was observed to be narrower in the vertical than in the normal development pattern. The nasopharynx in patients with class II malocclusions and vertical development patterns is substantially narrower than in patients with class II malocclusions and normal growth patterns [9].

De Freitas et al. conducted a study and reported that the upper pharyngeal width was statistically substantially narrower in participants with class I and class II malocclusions and vertical development patterns than in subjects with normal growth patterns [10]. Wang et al. found a reduced airway space in skeletal high-angle class II patients in their investigation on adults, confirming a link between pharyngeal airway space and a vertical skeletal pattern. He partially proved that a vertical growth pattern may predispose a person to pharyngeal narrowing, which may lead to upper airway blockage [11]. Ponnada et al. discovered that nasopharyngeal linear and angular measurements were lower in class II vertical growers than in class I ordinary growers. The nasopharyngeal region was also shown to be smaller in class II vertical growers [12].

As mandibular retrognathism is one of the most confounding factors responsible for skeletal class II malocclusion, studies have established a favorable link between temporomandibular disorders (TMDs) and abnormal morphology of the mandible [13]. The temporomandibular joint is a complicated anatomical component in the human body with substantial therapeutic consequences in dentistry. The condyle is a component of the temporomandibular joint complex, and its volume and form influence the treatment response and stability in orthodontic, orthopedic, orthognathic, and prosthodontic patients. As a result, dental practitioners should evaluate the position and morphology of the condyle during the treatment procedure.

Position of the Condyle in Class II Malocclusions

Earlier, it was assumed that only class III malocclusions are at a risk for TMD development; however, due to altered disc and condylar locations, even class II instances were considered high risk for TMD development [14,15]. Some authors discovered class II horizontal instances with increased TMD risk due to retruded incisors locking the mandible and changing functional connections. In contrast, class II vertical instances were found to be related to TMDs due to altered condylar position and disc modifications in the form of an anteriorly displaced disc, resulting in reduced TMJ stability [16,17]. Ricketts proved that the condyle position in class II division I type malocclusions was at an anterior position in the fossa prior to treatment [18]. Following therapy, the condyles returned to their usual position. The initial forward condylar position in these patients was described as an attempt to preserve an adequate airway. Ricketts discovered that in class II division 1 instances, both the condyles and the course of closure of the mandible showed considerably more distal migration from the rest position to centric occlusion than in class II division 2 cases using laminagraphy.

The vertical skeletal dimension is critical in treatment planning. Vertical skeletal patterns are associated with condylar location and morphology. Condylar size and shape are related to mandibular divergence in young adults [19]. Individuals with hyperdivergent development patterns have smaller superior joint spaces and more posteriorly inclined condyles, whereas those with normal or hypodivergent growth have larger superior joint spaces and more anteriorly slanted condyles [20]. Smaller anteroposterior and mediolateral condyle widths, as well as a smaller angle between the condylar axis and the mid-sagittal plane, are related to hyperdivergent face morphology. These data imply that condylar location and shape differ depending on vertical face morphology [21].

John et al. conducted a study on class II malocclusion vertical cases and discovered the greatest changes in disc position, condylar position, and joint gaps. When compared to class II horizontal cases, there was a predisposition for anterior and medial disc displacement with more anteriorly positioned condyles. Class II instances, particularly vertical cases, must be thoroughly investigated for the existence of TMD signs and symptoms, and if clinical examination yields positive results, the cases should be referred to an MRI [22]. Basafa et al. conducted a study to assess the prevalence of TMD in class II and class II malocclusion and found that it was more prevalent in class II malocclusion [23].

Treatment

Correction of the skeletal discrepancy is best achieved during active growth periods. Early treatment advocates argue that correcting skeletal discrepancies is just as beneficial in preadolescence as it is in adolescence. Other orthodontists believe that treatment should be delayed until the adolescent has reached the growth spurt occurs. In spite of the approach, it is important to recognize that clinically significant mandibular growth spurts do not occur in the majority of people [24]. The following are indicators that therapy should begin in class II malocclusions: Treatment for mild to moderate dental or skeletal irregularities could be delayed until the late mixed or early permanent dentition periods have been achieved. When there are more serious disparities, treatment can begin as soon as the individual is willing to participate or endure wearing the appliance. In these extreme situations, the doctor wishes to increase the possibility of rectifying the skeletal disparity while minimizing the possibility of damaging the projecting maxillary incisors. "Functional treatment is also provided with acceleration methods to shorten the treatment time in growing patients."

Correction of Class II in Growing Patients

There have been numerous appliances that are used for the correction of the developing class II malocclusions, including the following: (1) Orthopedic Hawley: In the mixed dentition, this appliance is used to treat class II division I malocclusions with extra-oral traction and a removable maxillary Hawley appliance with a labial bow on the anterior teeth. Around the banded first molars, circumferential clasps are placed to reduce distal movement caused by extra-oral force. If necessary, an anterior bite plate can be inserted into the Hawley retainer to help with the anteroposterior correction while also improving the deep overbite [25]. Finger springs might be used to treat localized dental issues, such as a single-tooth crossbite, or an expansion screw could be used to correct segmental posterior crossbites. (2) Extra-oral traction, in conjunction with a trans palatal arch between the first molars, can be utilized to reduce the distal mobility of these teeth in order to maximize the orthopedic effect on the maxilla. The advantage of this strategy is that patient cooperation is restricted to wearing the headgear; the negative is that the appliance incorporates fewer maxillary teeth, which reduces the possibility of an orthopedic effect.

Correction of Class II in Adults

Extraction of premolars is the most commonly used method of addressing dental and mild skeletal abnormalities in a class II malocclusion. Because orthodontic therapy cannot considerably modify the facial skeletal connection in adults, the extraction of upper first premolars will allow for the correction of the overjet while keeping the class II molar relationship. A fundamental assumption in such a treatment strategy is that the lower arch may be straightened and leveled without removing teeth. Essentially, dental compensations are used to conceal a small skeletal difference. Extraction of premolars in the lower arch for alignment of a severely crowded dentition and retraction of protrusive incisors will not correct class II correction unless some of the extraction space is used to protract the mandibular molars. If the mandibular dentition is moderately crowded, extraction of the second premolars and protraction of the first molars will aid in molar relationship repair. In class II division 1 cases with severe discrepancies in skeletal type, however, extractions in the lower arch are frequently contraindicated because any uprighting of the lower incisors increases the distance that the upper front teeth must be retracted to correct the overjet.

Treatment considerations for TMDs

Myalgia is the most common symptom and is a subset of temporomandibular disorder (TMD), which includes temporomandibular joint and associated muscular abnormalities. There is a wide range of low-cost and reversible treatments available for masticatory muscle problems. Treatments commonly used include education, stretching exercises, manual therapy, acrylic splints, and cognitive behavioral therapy. Self-exercise with occlusal splints has been shown to lessen pain symptoms and increase maximum mouth opening (MMO) and range of motion (ROM), with self-exercise with or without occlusal splints causing a significant reduction in discomfort [26].

TMD is currently most commonly treated with occlusal splints, a reversible therapy aimed at lowering TMJ load and, as a result, clinical symptoms. Ferrario et al. devised and used a splint with solely posterior teeth (molars and premolars) contacts; anterior teeth (incisors and canines) contacts, according to their biomechanical model, can enhance temporal muscle activity and, thus, the load on the TMJ [27]. The splint was created to promote left-to-right muscular balance and to prevent the consequences of muscular torque. Surface electromyography (EMG) has been used in various investigations because it allows for the verification and quantification of muscular balance on both sides of the body (symmetry) and between pairs of muscles with a potential torque influence on the mandible. Furthermore, the quantitative evaluation of muscle contraction patterns during dynamic standardized activities enables the measurement of neuromuscular coordination before and after occlusal surface change [28].

For almost 60 years, proliferative injection treatment (prolotherapy), also known as regenerative injection therapy (RIT), has been utilized to improve tendon, ligament, and joint repair [29]. The basic procedure of prolotherapy entails injecting medication into tendon and ligament insertions to encourage fibrous tissue proliferation in order to heal and stabilize the fibro-osseous junction (FOJ). TMJ prolotherapy, like other joint treatments, can be beneficial to individuals who have a temporomandibular disorder (TMD) that is resistive to or has demonstrated only limited improvement with physical medicine, dietary restrictions, and home care. It can also benefit patients who have not improved sufficiently with oral appliances, or who are unable or unable to wear such equipment, and who are unsuitable or unwilling candidates for TMJ surgery.

Among the non-surgical treatments, low-level laser therapy (LLLT) has recently received attention due to its ease of use, short treatment time, and lack of contraindications. In theory, LLLT is a non-thermal type of light that is expected to alleviate inflammation by inhibiting prostaglandin 2 (PEG2) production and suppressing cyclooxygenase 2 [30,31]. TMDs have been treated using a variety of wavelengths, the most popular of which is located in the electromagnetic spectrum between 780 and 904 nm [32]. Shirani et al. discovered that pain reduction remained statistically significant during this time period [33]. Mazzetto et al. discovered that the least discomfort to palpation occurred during the final session of laser application [34]. Ferreira et al. saw substantial effects in both pain reduction and functional outcomes after following patients for three months [35].

## Conclusions

Class II malocclusion can be accompanied by an anteroposterior skeletal disparity between the upper jaw and lower jaw, commonly with retrusion of the lower jaw, but the upper jaw can also protrude. The correlations are placed on a vertical facial pattern that varies between enhanced, normal, or decreased total and lower anterior facial heights. The position of the condyle also influences an individual's growth, causing TMJ impairment as well as airway obstruction. This is most common in class II malocclusion, and TMD treatment must begin as soon as imparity is detected.
